# Rapidly Progressive Atrioventricular Block in a Patient with Sarcoidosis

**DOI:** 10.1155/2014/372936

**Published:** 2014-08-21

**Authors:** Nagham Saeed Jafar, Warkaa Al Shamkhani, Sunil Roy Thottuvelil Narayanan, Anil Kumar Rajappan

**Affiliations:** Department of Cardiology, Belhoul Speciality Hospital, P.O. Box 5527, Dubai, UAE

## Abstract

Cardiac sarcoidosis is a major cause of death in patients with systemic sarcoidosis. Cardiac manifestations are seen in 2.3% of the patients. Atrioventricular (AV) block is one of the common manifestations of cardiac sarcoidosis. Other presentations of cardiac involvement include congestive heart failure, ventricular arrhythmias, and sudden cardiac death. The presence of AV block in young patients should raise the suspicion of sarcoidosis. AV block may be the only manifestation and patients may not have clinical evidence of pulmonary involvement. Here we describe a young male presented with exercise induced AV block rapidly progressing to complete heart block with recurrent syncope needing urgent pacemaker implantation. Factors that suggested an infiltrative process included his young age, rapidly progressive conduction abnormalities in the ECG in the absence of coronary disease, and previous history of cutaneous sarcoidosis.

## 1. Introduction

Sarcoidosis is a multisystem disease of unknown aetiology characterized by the formation of noncaseating granulomas in many tissues. The granulomas mostly affect the lungs and lymph nodes [1]. Cardiac manifestations are seen in 2.3% of the patients. The organ involvement is variable according to race, sex, and age with race appearing to be a major determinant factor [2]. Cardiac involvement is largely unrecognized. Cardiac sarcoidosis may be the presenting manifestation of sarcoidosis or may be asymptomatic. Atrioventricular block is one of the common manifestations of cardiac sarcoidosis, and it is a major cause of death in patients with systemic sarcoidosis. Other presentations of cardiac sarcoidosis include congestive heart failure (CHF), ventricular arrhythmias, and sudden cardiac death (SCD) [3]. A high index of suspicion is required as these patients can present initially only with arrhythmias and with no pulmonary involvement.

## 2. Case Report

47-year-old male presented to the cardiology out-patient department (OPD) with shortness of breath and chest discomfort on exertion since one week. Chest discomfort was related to exertion and relieved by rest. He has hypertension and hypertriglyceridemia controlled well on oral Amlodipine and Fenofibrate since one year. He does not smoke, drink alcohol, or use any recreational drugs. The patient gives past history of cutaneous sarcoidosis which was diagnosed by biopsy of the lesion one year back. He was evaluated for systemic sarcoidosis and was found to have hilar lymph nodes by computed axial tomography of chest and was advised for a follow-up CT after 6 months. The follow-up CT scan showed improvement and the patient was asymptomatic. Corticosteroids were not started at that time as the patient was concerned about the side effects of steroids on longterm. Hence he was advised for regular follow up. Three months after this evaluation, he presented to our OPD with the present symptoms. Physical examination did not show cutaneous lesions, his pulse was regular at 62 beats per minute and the blood pressure was normal. Cardiovascular examination revealed normal S1 and S2 with no gallop, murmurs, or rub.

**Figure 1:**
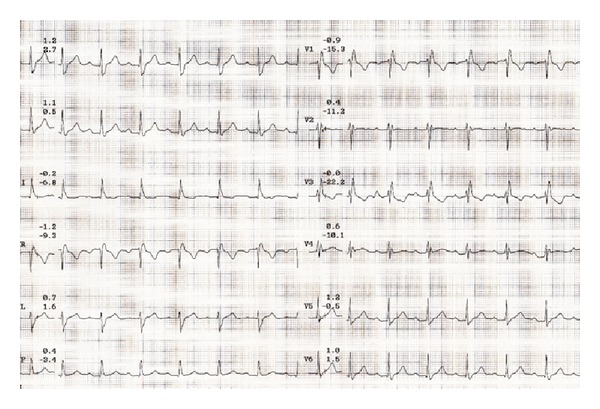
Baseline electrocardiogram showing sinus rhythm with right bundle branch block (RBBB).

**Figure 2:**
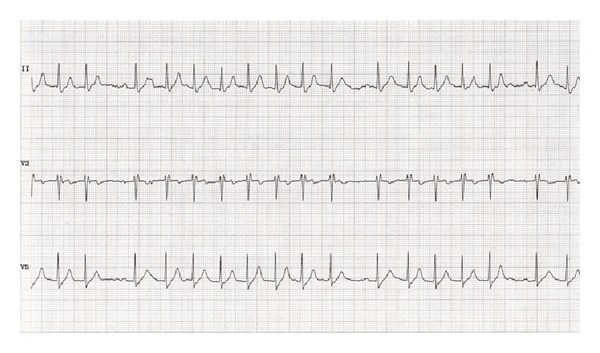
ECG during exercise test showing intermittent high grade AV block.

**Figure 3:**
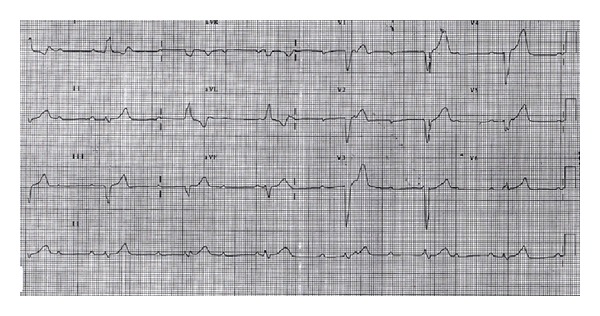
Electrocardiogram showing complete heart block (CHB). Ventricular rate is 25 per minute and QRS complex is very wide and there is AV dissociation.

**Figure 4:**
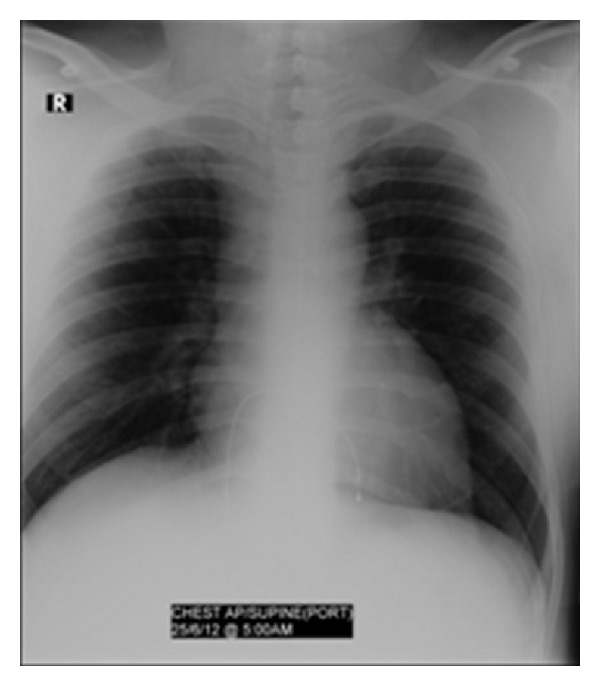
CXR shows cardiac enlargement and pulmonary venous congestion. Temporary pacing lead is seen positioned in the right ventricle. There is no hilar lymph adenopathy.

At the time of presentation his electrocardiography (ECG) revealed sinus rhythm with a rate of 62 per minute with prolonged PR interval and right bundle branch block (RBBB) (Figure 1). Cardiac enzymes were normal. He has undergone echocardiography and treadmill exercise test for the evaluation of his chest discomfort. His echocardiography showed normal left and right ventricular function without any regional wall motion abnormalities. His stress test showed poor chronotropic response and rate dependent 2 : 1 atrioventricular (AV) block occurring during the exercise test (Figure 2). Exercise was terminated at 3.5 METS due to severe fatigue and second degree AV block in ECG. The ECG reverted to sinus rhythm in the recovery phase. He was taken up for a coronary angiography next day which showed normal coronaries. He was discharged from the hospital after coronary angiography but he presented 3 days later to the ER with recurrent episodes of syncope. His ECG showed complete heart block (CHB) (Figure 3), with low ventricular rate and wide QRS complex. Chest X-ray does not show any hilar lymphadenopathy but showed cardiomegaly and pulmonary venous congestion (Figure 4).

He has undergone urgent temporary pacemaker implantation. This was followed by permanent pacemaker implantation with Victory XL DDDR pacemaker (St. Jude Medical). He made an uneventful recovery. He remained asymptomatic afterwards. A workup for the autoimmune profile was normal, serum calcium was 8.6 mg/dL, and the serum angiotensin converting enzyme level was elevated. Pacemaker interrogation showed he is fully dependent on pacemaker at 2 weeks after discharge. He remained symptom-free following the procedure. He was evaluated by an internist and a dermatologist after the surgical wound was healed. Based on the clinical presentation and available reports he was started on immunosuppressive therapy with steroids. Presently his ECG shows sinus rhythm with RBBB and pacemaker on demand mode. He is doing well one year after the procedure.

## 3. Discussion

Cardiac sarcoidosis is a major cause of death in patients with systemic sarcoidosis. Clinically evident sarcoidosis involving the heart affects 2.3% of patients [3]. But autopsy studies have shown the incidence to be between 20 and 25% [4]. Common presentations of cardiac sarcoidosis include congestive heart failure, cardiac rhythm disturbance, and sudden death. Incidence of sudden cardiac death is about 20%, with the terminal rhythm being high grade AV block or ventricular tachycardia/fibrillation [5].

Cardiac involvement can occur at any point during the course of sarcoidosis. Asymptomatic heart involvement is more common in systemic sarcoidosis. When symptomatic, cardiac involvement can present as CHF, AV block, cardiac arrhythmias, and SCD. The clinical presentation of cardiac sarcoidosis reflects the changes in myocardium caused by granulomatous deposition leading to inflammation and subsequently scar. These become the source of ventricular arrhythmias and conduction block.

In a prospective study of 32 patients aged less than 60 years presenting with CHB, 34% had previously undiagnosed cardiac sarcoidosis. This group had higher adverse cardiac events compared with patients without sarcoidosis [6]. Advanced AV block in young patients should raise the suspicion of sarcoidosis if it is associated with CHF or arrhythmias. Isolated AV block can occur with normal left ventricular function when the diagnosis of cardiac sarcoidosis can be challenging.

The prognosis of symptomatic cardiac sarcoidosis is not clear, but 5-year mortality rates exceed 50% [7]. Incidence of cardiac sarcoidosis may be higher than expected in patients with idiopathic AV block. The myocardial involvement occurs in 25% of patient. When extensive area of myocardium is involved, patient presents with CHF or ventricular arrhythmias. The usual predilection of the disease is the anterior basal interventricular septum leading to the conduction abnormalities.

Reaching a correct diagnosis early in the course of the disease is difficult unless sarcoidosis is also present in other organs. Early administration of immunosuppressant therapy (high dose steroids) may stop or reverse cardiac damage [8]. Our patient has presented with cardiac symptoms which deteriorated rapidly to CHB. Even though the diagnosis of cardiac sarcoidosis is clinical and mostly presumptive, earlier tissue diagnosis of cutaneous sarcoid by biopsy and pulmonary sarcoid by CT scan makes cardiac sarcoid as the reason for his CHB.

An accurate and early diagnosis is essential to enable prompt treatment of arrhythmias. The recovery of his rhythm back to sinus after the institution of steroid also justifies the presumptive diagnosis of cardiac sarcoidosis in this patient. Early institution of steroid therapy is advised in patient with systemic sarcoidosis to prevent fatal cardiac complication even if they are asymptomatic.
